# Evaluation of circadian phenotypes utilizing fibroblasts from patients with circadian rhythm sleep disorders

**DOI:** 10.1038/tp.2017.75

**Published:** 2017-04-25

**Authors:** A Hida, Y Ohsawa, S Kitamura, K Nakazaki, N Ayabe, Y Motomura, K Matsui, M Kobayashi, A Usui, Y Inoue, H Kusanagi, Y Kamei, K Mishima

**Affiliations:** 1 Department of Psychophysiology, National Institute of Mental Health, National Center of Neurology and Psychiatry, Tokyo, Japan; 2 Japan Society for the Promotion of Science, Tokyo, Japan; 3 Yoyogi Sleep Disorder Center, Tokyo, Japan; 4 Department of Somnology, Tokyo Medical University, Tokyo, Japan; 5 Department of Neuropsychiatry, Bioregulatory Medicine, Akita University, Graduate School of Medicine, Akita, Japan; 6 Department of Laboratory Medicine, National Center Hospital, National Center of Neurology and Psychiatry, Tokyo, Japan

## Abstract

We evaluated the circadian phenotypes of patients with delayed sleep–wake phase disorder (DSWPD) and non-24-hour sleep–wake rhythm disorder (N24SWD), two different circadian rhythm sleep disorders (CRSDs) by measuring clock gene expression rhythms in fibroblast cells derived from individual patients. *Bmal1-luciferase* (*Bmal1*-*luc*) expression rhythms were measured in the primary fibroblast cells derived from skin biopsy samples of patients with DSWPD and N24SWD, as well as control subjects. The period length of the *Bmal1*-*luc* rhythm (*in vitro* period) was distributed normally and was 22.80±0.47 (mean±s.d.) h in control-derived fibroblasts. The *in vitro* periods in DSWPD-derived fibroblasts and N24SWD-derived fibroblasts were 22.67±0.67 h and 23.18±0.70 h, respectively. The N24SWD group showed a significantly longer *in vitro* period than did the control or DSWPD group. Furthermore, *in vitro* period was associated with response to chronotherapy in the N24SWD group. Longer *in vitro* periods were observed in the non-responders (mean±s.d.: 23.59±0.89 h) compared with the responders (mean±s.d.: 22.97±0.47 h) in the N24SWD group. Our results indicate that prolonged circadian periods contribute to the onset and poor treatment outcome of N24SWD. *In vitro* rhythm assays could be useful for predicting circadian phenotypes and clinical prognosis in patients with CRSDs.

## Introduction

Circadian rhythm sleep disorders (CRSDs) are defined by persistent or recurrent disturbed sleep–wake patterns and consist of several subtypes including advanced sleep–wake phase disorder (ASWPD), delayed sleep–wake phase disorder (DSWPD), and non-24-hour sleep–wake rhythm disorder (N24SWD).^1, 2, 3, 4^ ASWPD is characterized by extremely early involuntary sleep timing, whereas DSWPD is characterized by significantly delayed sleep timing, and N24SWD has sleep timing that occurs with a 30 min to 1 h delay each day. CRSDs have a high rate of comorbidity with various psychiatric disorders, especially mood disorders.^5, 6, 7, 8^ A previous study showed that approximately half of their patients with N24SWD developed psychiatric problems before or after the onset of N24SWD.^7^ CRSDs are thought to result from impairment of the circadian clock system and/or a misalignment between the endogenous circadian rhythm and exogenous entrainment factors that affect sleep timing. Patients with CRSDs are mostly treated with chronotherapy, in which intense light exposure or melatonin administration is performed during the phase-advance or phase-delay portion, respectively, of the sleep–wake cycle. In mammals, the central oscillator in the suprachiasmatic nucleus of the hypothalamus incorporates environmental cues, such as light exposure, and coordinates the phase of oscillators in peripheral tissues.^9, 10^ The molecular mechanisms underlying the circadian clock system involve the transcription–translation negative feedback loops of multiple clock genes including *BMAL1*, *CLOCK*, *CRY*, *PER*, *ROR* and *REV-ERB*.

Evaluating the circadian phenotype is crucial for establishing a precise clinical diagnosis and for understanding the pathophysiology of diseases that are associated with disturbed biological rhythms such as CRSDs. The intrinsic circadian period, *τ* (the free-running period of circadian rhythms in the absence of external cues), is considered to be a critical factor in the pathophysiology of CRSDs.^1, 2, 3, 4^ In fact, we found that the *τ* determined under a forced desynchrony protocol was longer in patients with N24SWD than it was in healthy subjects with an intermediate chronotype.^11^ However, the forced desynchrony protocol is costly and laborious,^12, 13^ and thus different approaches that can provide more convenient and feasible methods of evaluating circadian phenotypes in a clinical setting are needed.

Surrogate measurement techniques, such as using cultured cells derived from biopsy samples of an individual, have been developed and tested for assessing circadian phenotypes.^14, 15, 16, 17, 18, 19^ We recently measured clock gene expression rhythms (*in vitro* rhythms) in primary fibroblasts obtained from skin biopsy samples of healthy subjects and compared the period length of *in vitro* rhythms (the *in vitro* period) with the subjects' circadian/sleep parameters, as evaluated by questionnaires, sleep logs and actigraphy.^14^ The results showed that the *in vitro* period was significantly correlated with subjects' chronotypes and habitual sleep time. Our data suggest that evaluating the *in vitro* period may be useful for predicting circadian phenotypes. However, the approach of using isolated cultured cells has not yet been applied to assess patients with CRSDs. Therefore, in the present study, we examined the circadian phenotypes of patients with DSWPD and N24SWD by evaluating *Bmal1*-*luciferase* (*Bmal1*-*luc*) rhythms in skin fibroblast cells from individual patients.

## Materials and methods

### Subjects

The study population consisted of 41 individuals with DSWPD (29 men; mean±s.d. age: 32.14±9.86 years and 12 women; mean±s.d. age: 33.08±13.28 years), 26 individuals with N24SWD (17 men; mean±s.d. age: 28.82±8.60 years and 9 women; mean±s.d. age: 30.33±14.44 years) and 50 controls (50 men; mean±s.d. age: 27.06±7.42 years; Supplementary Tables S1–S3). All subjects were recruited at medical and research institutes on mainland Japan and were sighted individuals. The patients with DSWPD and N24SWD were clinically diagnosed by trained psychiatrists according to the International Classification of Sleep Disorders, 2nd Edition.^20^ Controls were healthy individuals with intermediate chronotypes (mean±s.d. morningness–eveningness questionnaire (MEQ) score: 50.89±4.04). The Japanese version of the Horne–Östberg MEQ was used to assess control subjects' chronotypes.^21^ Because an individual's morningness–eveningness preference changes with age,^22^ the MEQ scores were adjusted by age (age-adjusted MEQ score: MEQ score+0.3512 × [39.212−age]).^23^ The protocol was approved by the Institutional Ethics Committee of the National Center of Neurology and Psychiatry, and written informed consent was obtained from all the subjects. The present study was conducted according to the principles of the Declaration of Helsinki.

### Skin biopsy, cell culture and *in vitro* rhythm assay

**Figure 1:**
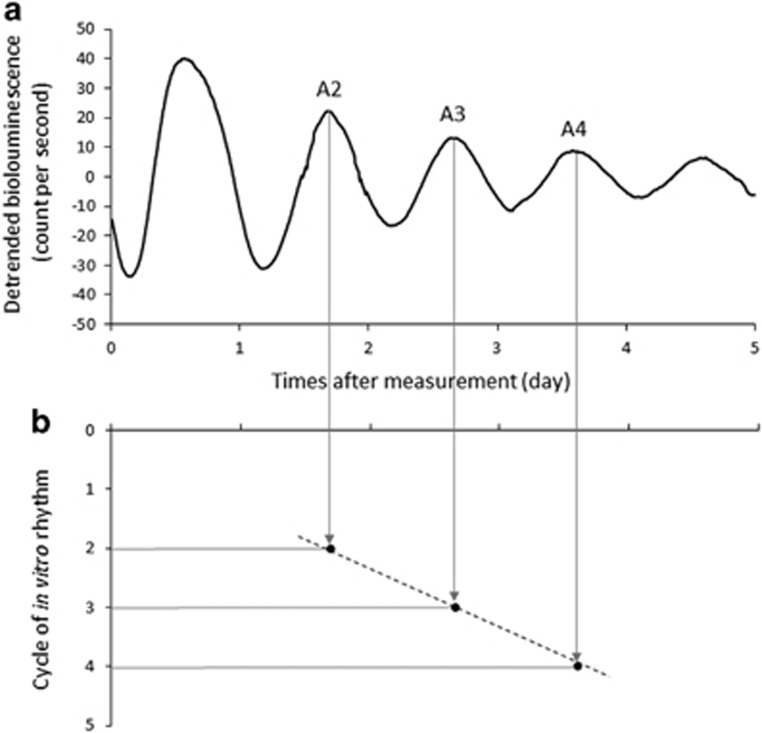
Representative detrended data of *Bmal1*-*luc* rhythm (*in vitro* rhythm) in cultured fibroblasts from a control subject. (**a**) A linear regression line (dotted line) was calculated using acrophase times for the second cycle (A2), third cycle (A3) and fourth cycle (A4) of the *in vitro* rhythm. (**b**) The period length of the *Bmal1*-*luc* rhythm (*in vitro* period) was determined from the slope of the regression line.

The experimental procedures were performed according to our previous study.^14^ Briefly, for each measurement, 1 × 10^6^ primary fibroblast cells derived from a skin biopsy sample were transfected with 5 μg of the *Bmal1*-*luc* reporter construct Bp/527-LUC^24^ using Neon (Thermo Fisher Scientific, Waltham, MA, USA) and were plated in a 35 mm culture dish. After 14 days, the cells were treated with 0.1 μm dexamethasone (Sigma-Aldrich, St. Louis, MO, USA) for 2 h to synchronize the rhythms in the fibroblasts. Luminescence from the cells was measured in recording medium using a LumiCycle (Actimetrics, Wilmette, IL, USA). The period length of the *Bmal1*-*luc* rhythm (*in vitro* period) was determined by regression analysis as previously reported.^14, 25, 26, 27^ The luminescence data were detrended by subtracting the 24 h moving average from the raw data and then was smoothed by 2 h adjacent average. The acrophase of the *in vitro* rhythm was calculated using ClockLab (Actimetrics). A linear regression line was determined using acrophases for the second, third and fourth cycles of the *in vitro* rhythm. The slope of the regression line indicates the *in vitro* period (Figure 1). The *in vitro* period for each subject was determined as the mean of three to six independent measurements. The *in vitro* periods in the control, DSWPD and N24SWD groups are presented as mean±s.d.

### Chronotherapy and treatment response

Chronotherapy consisted of high-intensity light therapy and the administration of melatonin or a melatonin receptor agonist (ramelteon) as previously described.^28^ Less than 6 h after waking up, the patients were exposed to high-intensity light (5000–8000 lx) for 2–3 h. The patients took 1, 0.5 and 0.5 mg of melatonin 7, 5.5 and 4 h, and took 4 mg of ramelteon 7 h, before going to bed on the previous day. The patients kept their sleep diaries during the therapy. Mid-sleep time was designated as the midpoint between sleep-onset time and wake time. Patients with DSWPD were considered to have responded to the therapy if their mid-sleep times during the therapy were entrained to their desired times for four consecutive weeks. The circadian period (*τ*) of the sleep–wake cycle was calculated by performing linear regression analysis using mid-sleep times as previously described.^11^ Patients with N24SWD were considered to have responded if the *τ* of the 4-week sleep–wake cycle during the therapy was 24.1 h or less, as described for the tasimelteon clinical trials.^28^ Mid-sleep time and *τ* are presented as mean±s.d.

### Statistical analysis

**Figure 2:**
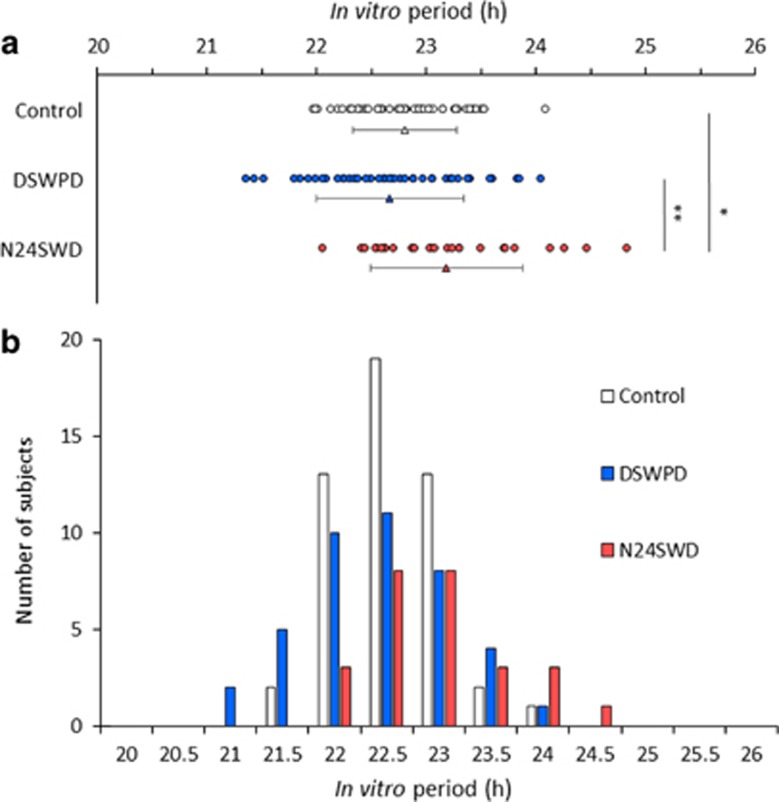
The period length of *Bmal1-luc* rhythm (*in vitro* period) in the control, DSWPD and N24SWD groups. (**a**) *In vitro* periods in 50 control fibroblast samples (open circle), 41 DSWPD fibroblast samples (blue circle) and 26 N24SWD fibroblast samples (red circle). Triangles represent mean *in vitro* periods in the control group (open), DSWPD group (blue) and N24SWD group (red). Data are presented as mean±s.d.; *P<0.05; **P<0.01. (**b**) Frequency distribution of *in vitro* period in control fibroblast samples (open bar), DSWPD fibroblast samples (blue bar) and N24SWD fibroblast samples (red bar). Each bin represents a 0.5 h period. DSWPD, delayed sleep–wake phase disorder; N24SWD, non-24-hour sleep–wake rhythm disorder.

**Figure 3:**
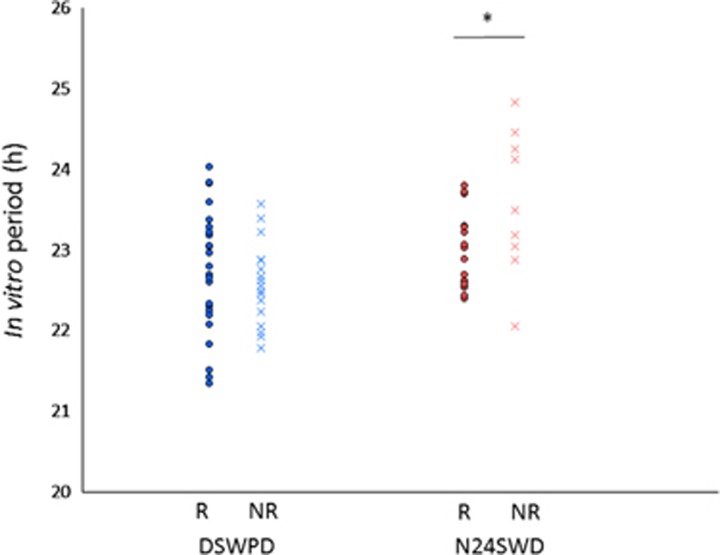
The period length of *Bmal1-luc* rhythm (*in vitro* period) in the responders (circle) and non-responders (cross) from the DSWPD (blue) and N24SWD (red) groups. No significant difference was observed in the *in vitro* period between the responders (R) and non-responders (NR) in the DSWPD group. Longer *in vitro* period was observed in the non-responders (NR) compared with the responders (R) in the N24SWD group. *One-tailed *P*<0.05. DSWPD, delayed sleep–wake phase disorder; N24SWD, non-24-hour sleep–wake rhythm disorder.

Kolmogorov–Smirnov tests were performed and frequencies for the parameters tested in this study were normally distributed. Levene's tests were performed and Welch's correction for unequal variances was applied to *t*-test. One-way analyses of variance and Bonferroni *post hoc* testing were performed to compare the *in vitro* periods among the DSWPD, N24SWD and control groups (Figure 2). One-tail unpaired *t*-tests were used to compare the *in vitro* period of the responders with that of the non-responders in each of the DSWPD and N24SWD groups (Figure 3). *P*<0.05 was considered to be statistically significant. Data analysis was performed using ORIGIN9 (OriginLab, Northampton, MA, USA).

## Results

The *Bmal1-luc* rhythms were measured in the fibroblast cells derived from patients with DSWPD, patients with N24SWD and control subjects. The period length of the *Bmal1*-*luc* rhythm (*in vitro* period) varied among individuals. The *in vitro* period ranged from 21.35 to 24.04 h (mean±s.d.: 22.67±0.67 h) in DSWPD fibroblast samples, from 22.05 to 24.83 h (mean±s.d.: 23.18±0.70 h) in N24SWD fibroblast samples and from 21.96 to 24.08 h (mean±s.d.: 22.80±0.47 h) in control fibroblast samples (Figure 2). The *in vitro* period differed significantly among the three groups (F(2,114)=4.37, *P*=0.003). A prolonged *in vitro* period was observed in the N24SWD group compared with the control (Bonferroni-corrected *P*=0.029) or DSWPD group (Bonferroni-corrected *P*=0.002). In contrast, no difference in the *in vitro* period was observed between the DSWPD and control groups.

Patients with CRSDs underwent chronotherapy. In some patients, the CRSD was intractable and they were considered to be non-responders. The relationship between treatment response and *in vitro* period were assessed in the DSWPD and N24SWD groups (Table 1 and Figure 3). The *in vitro* period did not differ between the responders and the non-responders in the DSWPD group (22.73±0.77 h vs 22.58±0.51 h; *t*=0.69, df=39, one-tailed *P*=0.246) but did differ in the N24SWD group (22.97±0.47 h vs 23.59±0.89 h; *t*=−1.95, df=10.42, one-tailed *P*=0.039 (Welch correction)). The *in vitro* period of the non-responders was longer than that of the responders in the N24SWD group. Detailed information of the control subjects and patients is shown in Supplementary Tables S1–S3.

## Discussion

To our knowledge, this is the first study to assess the circadian phenotypes of patients with CRSDs by performing surrogate measurements using fibroblast cells in culture. Our data showed that the *in vitro* period was significantly longer in the N24SWD group than it was in the control group. These findings are consistent with our previous study, which demonstrated that the *τ* of the melatonin rhythms in patients with N24SWD was longer than the *τ* in control subjects.^11^ Our *in vitro* and *in vivo* findings demonstrating a longer circadian period in the N24SWD group strongly support the notion that prolongation of the circadian period contributes to the N24SWD phenotype.

Theoretically, a long *τ* delays the phase of circadian rhythms.^13^ A significantly longer *τ* would result in continuous phase delays in the sleep–wake cycle, which is the typical phenotype of N24SWD. However, a prolonged *in vitro* period was not necessarily observed in all of the N24SWD fibroblast samples. This indicates that other factors have a role in the N24SWD phenotype. For instance, impaired photic entrainment of the circadian clock is considered to be one factor that leads to the onset of N24SWD, as nearly 50% of completely blind individuals show free-running sleep–wake patterns.^1^ In contrast, the *in vitro* periods of the DSWPD and control groups did not differ. This finding suggests that circadian period length is not a primary factor in patients with the DSWPD phenotype. It has been proposed that alterations in circadian entrainment mechanisms could lead to the onset of DSWPD.^17^ Possible altered mechanisms include reduced phase advance and/or enhanced phase delay of the sleep–wake cycle and decreased phase-advance portion and/or increased phase-delay portion of the sleep–wake cycle.

In accordance with the results of previous reports,^11, 28, 29, 30^ our patients with CRSDs varied in their responses to chronotherapy. Notably, the chronotherapy non-responders had a longer *in vitro* period compared with the responders. This finding suggests that longer *in vitro* period might predict poorer treatment response in patients with N24SWD. McCarthy *et al.*^31^ previously measured *in vitro* rhythms in the fibroblast cells from bipolar disorder patients and examined the effect of the mood stabilizer lithium on *in vitro* rhythms. They found prolonged period length of *in vitro* rhythm and reduced responsiveness to lithium in bipolar disorder fibroblast samples. The relationship between *in vitro* period length and clinical response to lithium treatment was not assessed as the clinical data were not available in their study. Pharmacological assays using isolated fibroblasts might be useful for evaluating responsiveness to therapeutic agents and impairment of circadian entrainment mechanism in patients with CRSDs.

Previous studies have generally shown that *in vitro* rhythms are not strongly correlated with *in vivo* rhythms such as the melatonin-secretion rhythm.^14, 15, 17^ Nevertheless, correlations between fibroblast period and human daily behavior have been observed.^14, 18, 19^ The cultured cells are dissociated from other tissues and are isolated from any external and internal circadian signals. By contrast, *in vivo* tissues are co-dependent and interact closely with one another. It has been suggested that *in vitro* rhythms reflect the molecular mechanisms of the circadian oscillators in peripheral cells, whereas *in vivo* rhythms reflect the physiological mechanisms of the circadian clock system of an individual. In addition, a missense mutation in the *PER2* gene has been identified in a large pedigree with familial ASWPD.^32, 33^ Vanselow *et al.*^34^ demonstrated that altered *in vitro* rhythms were observed in fibroblast cells that express the *PER2* gene variant that has the same mutation found in the familial ASWPD pedigree. These findings indicate that *in vitro* rhythms represent intrinsic clock properties in cells and suggest that *in vitro* rhythm assays may be utilized for assessing individual genetic (but not physiological) differences in the circadian clock system. Further analyses using *in vitro* rhythm assays could provide new insights into the molecular pathology of CRSDs and may serve as a system that could be used to screen for potential therapeutic agents on a personalized basis.

There are some limitations to this study. Sex and age were not matched between patients with CRSDs and control subjects. It has been previously reported, though, that the *in vitro* period did not differ between young healthy subjects (11 men and 7 women; mean±s.d. age: 25.44±3.58 years) and old healthy subjects (11 men and 7 women; mean±s.d. age: 67.89±7.32 years).^16^ Thus, we believe that neither sex nor age would have influenced *in vitro* period. Responders and non-responders to chronotherapy were classified on the basis of *τ* of the sleep–wake cycle. Previous studies have, however, shown that sleep–wake rhythms do not always synchronize with the melatonin-secretion rhythm known as a circadian phase marker.^35^ Evaluating physiological rhythms as well as sleep–wake rhythms would be required to demonstrate that *in vitro* period could serve as a reliable molecular marker for the treatment outcome of CRSDs.

## Figures and Tables

**Table 1:** *In vitro* period and sleep pattern of responders and non-responders in DSWPD and N24SWD

	DSWPD			N24SWD		
	*N*	*In vitro* period (h)	Mid-sleep time (h)	*N*	*In vitro* period (h)^a^	*τ* (h)
Responders	24	22.73±0.77	3.90±1.06	17	22.97±0.47	23.94±0.07
Non-responders	17	22.58±0.51	5.97±1.12	9	23.59±0.89	24.48±0.37

Abbreviations: DSWPD, delayed sleep–wake phase disorder; N, number of subjects; N24SWD, non-24-hour sleep–wake rhythm disorder; *τ*, circadian period of the sleep–wake cycle during the chronotherapy.

a *P*<0.05 between responders and non-responders.

Mid-sleep time represents the midpoint between sleep-onset time and wake time during the chronotherapy. Data are presented as mean±s.d.
